# THE PROTECTIVE ROLE OF SOCIAL SUPPORT ON DEPRESSIVE SYMPTOMS AMONG AGING SEXUAL MINORITY CANADIANS

**DOI:** 10.1093/geroni/igz038.1113

**Published:** 2019-11-08

**Authors:** Arne Stinchcombe, Nicole G Hammond, Kimberley Wilson

**Affiliations:** 1 Saint Paul University (Ottawa), Ottawa, Ontario, Canada; 2 University of Ottawa, Ottawa, Ontario, Canada; 3 University of Guelph, Guelph, Ontario, Canada

## Abstract

Sexual minority older adults face minority stressors that are associated with higher rates of mental illness. The stress buffering effects of social support within majority populations are well documented. Using a large population-based sample of aging Canadians, we sought to examine the relationship between sexual orientation and depressive symptoms, and determine whether this relationship is moderated by social support and sex. Baseline data from the Canadian Longitudinal Study on Aging (CLSA) were used (n = 46147). Participants were between the ages of 45-85 years at time of recruitment (mean age = 62.46, SD = 10.27), and self-reported their sexual orientation as heterosexual or lesbian, gay, or bisexual (LGB) (2.1%). Social support and depressive symptoms were measured using validated instruments. Four functional social support subscales were derived: tangible, positive social interaction, affectionate, and emotional/informational. Multiple linear regression models adjusted for relevant covariates were conducted. LGB identification was associated with greater depressive symptoms when compared to heterosexual participants (p = 0.032). As evidenced by a significant 3-way interaction (p = 0.030), increasing tangible social support was associated with a corresponding decrease in the risk of depressive symptoms; this relationship was most pronounced for lesbian and bisexual women. A significant 2-way interaction (p = 0.040) revealed that as emotional/informational social support increased, depressive symptoms decreased, with greater disparity between LGB and heterosexual participants at lower levels of social support. The results highlight the importance of social support in promoting mental health, especially among sexual minority older adults.

